# Brain activation in older adults with hypertension and normotension during standing balance task: an fNIRS study

**DOI:** 10.3389/fnagi.2024.1458494

**Published:** 2024-09-24

**Authors:** Weichao Fan, Qing Zeng, Peng Zheng, Shuyang Wen, Gege Li, Tao Fan, Guozhi Huang, Manxu Zheng, Qinglu Luo

**Affiliations:** ^1^Department of Rehabilitation Medicine, Zhujiang Hospital, Southern Medical University, Guangzhou, China; ^2^School of Rehabilitation Medicine, Southern Medical University, Guangzhou, China; ^3^School of Nursing, Southern Medical University, Guangzhou, China; ^4^Department of Rehabilitation Medicine, The Tenth Affiliated Hospital of Southern Medical University (Dongguan People’s Hospital), Dongguan, China; ^5^Dongguan Experimental Centre for Sports Rehabilitation Research, Dongguan, China; ^6^Dongguan Key Specialty of Traditional Chinese Medicine (Rehabilitation Department), Dongguan, China

**Keywords:** aging, hypertension, balance, standing, posture control, fNIRS

## Abstract

**Background:**

Hypertension (HT) is a common chronic disease in older adults. It not only leads to dizziness and other symptoms affecting balance in older adults with HT but also affects the hemodynamics of the cerebral cortex. At present, potential neural mechanisms of balance control in older adults with HT are still unclear. Therefore, this study aimed to explore the differences in the center of pressure (COP) and cerebral cortex activation between older adults with HT and normotension (NT) during standing balance tasks. This study May provide guidance for the early detection of the risk of falls among older adults with HT and the development of clinical rehabilitation strategies.

**Methods:**

In this cross-sectional study, 30 older adults with NT (NT group) and 27 older adults with HT (HT group) were subjected to three conditions: task 1, standing with eyes open on a stable surface; task 2, standing with eyes closed on a stable surface; and task 3, standing with eyes open on the surface of the foam pad. Cortical hemodynamic reactions were measured using functional near-infrared spectroscopy, and COP parameters were measured using a force plate.

**Results:**

The mean velocity of the COP in the medial–lateral direction in the NT group was significantly higher than that in the HT group (*F* = 5.955, *p* = 0.018) during task 3. When proprioception was disturbed, the activation of the left premotor cortex and supplementary motor cortex in the HT group was significantly lower than that in the NT group (*F* = 14.381, *p* < 0.001).

**Conclusion:**

The standing balance function of older adults with HT does not appear to be worse based on COP parameters than those of older adults with NT. This study revealed that the changes in the central cortex related to standing balance appear to be more indicative of balance control deficits in older adults with HT than changes in peripheral COP parameters, suggesting the importance of the early evaluation of cortical activation in older adults with HT at risk of falls.

## Background

Hypertension (HT) is a common condition for older adults (Wang et al., 2023). In China, more than half of the older adult population live with HT, and 55.7% of people aged >65 years have HT (Chen et al., 2022). Of note, HT is one of the risk factors for falls (Buford, 2016; Guerra et al., 2021; Xu et al., 2022). The risk for falls among older adults with arterial HT is approximately seven times higher than that of older adults without this clinical condition (Guerra et al., 2021). Neurobiological mechanisms underlying worsening balance control must be elucidated, which will not only allow for the identification of fall-risk individuals, but also constitute the basis for developing targeted preventive and rehabilitative intervention strategies.

Previous studies have reported the relationship between HT and balance disorder (Bromfield et al., 2017; Bursztyn, 2017). Compared with healthy older adults, older adults with HT are more prone to instability, dizziness, and vertigo (Abate et al., 2009). A recent study has shown that individuals with HT show decreased static balance function and a higher fear of falls (Ozaldemir et al., 2020). However, the fall questionnaire lacks objectivity and has experimenter bias, whereas static posturography May be an index of the fall risk in older people. Sway parameters extracted from the center of pressure (COP) were found to correlate with both fall risk factors and deficient postural strategies (Quijoux et al., 2020). However, in biomechanical studies using COP, no difference in balance function was found between individuals with and without HT (Abate et al., 2009). Therefore, existing evidence concerning the balance control of older adults with HT remains conflicting.

Maintaining upright posture balance in a well-functioning nervous system mainly depends on the complex integration and coordination of several systems that cover cortical, subcortical, and spinal network control (Bolton, 2015). Cortical activity is involved in static balance control in older adults (Rubega et al., 2021; Solis-Escalante et al., 2019). In difficult balance tasks, the activations of the prefrontal cortex and supplementary motor area were related to age (Herold et al., 2017a; Herold et al., 2017b; Lehmann et al., 2022), and a drop in cerebral oxygenation in older adults on standing correlated with postural instability (Fitzgibbon-Collins et al., 2021). In addition, HT was found to be associated with reduced cerebral perfusion (Muller et al., 2012; Triantafyllou et al., 2020). These brain changes May affect the balance function of older adults with HT (Buford, 2016; Hausdorff et al., 2003; Yao et al., 2024). Although studies have unveiled significant age differences in brain responses during balance control in older adults, most studies have solely focused on healthy older adults (Lehmann et al., 2022; St et al., 2021). Few of them explored the potential neurobiological mechanisms of balance control in older adults with HT. Functional near-infrared spectroscopy (fNIRS) is a portable brain imaging technology, which can further clarify changes in the cerebral cortex and the neurophysiological mechanism involved in standing balance (Herold et al., 2017a; Malcolm et al., 2021). fNIRS has been used to explore cortical activation during postural tasks, and regions of interest have primarily covered the prefrontal cortex, the supplementary motor area, the primary motor cortex, the sensorimotor cortex (Herold et al., 2017b; Menant et al., 2020).

At present, potential neural mechanisms of balance control in older adults with HT are still unclear. In this study, we aimed to explore the differences in COP and cerebral cortex activation between older adults with HT and those with normotension (NT) during standing balance tasks. Compared with the NT group, we expect that the HT group will experience greater difficulty with the challenging standing tasks, resulting in the low activation of relevant brain control regions, particularly in motor-related cortical areas. This study May provide guidance for the early detection of older adults with HT at risk of falls and the development of clinical rehabilitation strategies.

## Methods

### Participants

This cross-sectional study used a moderate effect size of 0.3 (f), power of 0.80, *α*-level of 0.05, and correlation among the repeated measures of 0.4. In the power analysis, a minimum of 54 participants was required (at least 27 per group). In this study, a total of 57 older adults, including 30 in the NT group and 27 in the HT group, were recruited from the communities near the Zhujiang Hospital of Southern Medical University.

The inclusion criteria were as follows: (a) age ≥ 65 years, (b) HT diagnosis by a family doctor based on international guidelines defined as systolic blood pressure ≥ 140 mmHg and/or diastolic blood pressure ≥ 90 mmHg (Unger et al., 2020), (c) intake of antihypertensive drugs, and (d) absence of drugs that affect postural stability (e.g., sedative-hypnotics). The exclusion criteria were as follows: (a) unable to stand alone for 1 min as required by the experimental paradigm; (b) suffering from severe neurological, sensory, or muscular diseases (e.g., diabetes, Parkinson’s disease, peripheral neuropathy, cerebellar lesion, vestibular disease, mental illness, and vision or hearing impairment); (c) cognitive impairment according to Mini-Mental Status Examination (MMSE) score of <24 (Katzman et al., 1988) or inability to communicate normally; and (d) a history of knee or hip replacement surgery that affected standing performance. All participants signed the informed consent form.

The study protocol was approved by the Medical Ethics Committee of Zhujiang Hospital, Southern Medical University, Guangzhou, Guangdong, China (2022-KY-083-01).

### Clinical measurements

The participants’ general information such as age, height, weight and years of education were self-reported by the participants. Considering that the older adults have a variety of diseases, which May be a confounding factor affecting the results, participants were required to self-report the number of existing diseases (i.e., comorbidities), the existing diseases of the participants were added up as the total number of comorbidities. The MMSE scores of the participants were evaluated by experienced doctors.

The Berg balance scale (BBS) was used to assess the balance function of the participants, the reliability of the BBS was 0.97 (Downs, 2015). The activities-specific balance confidence scale (ABC) was used to measure the balance confidence of the participants, the reliability of the Chinese version of the ABC was 0.98 (Guan et al., 2012). Depression symptoms were screened using the Center for Epidemiological Studies Depression Scale (CES-D), the reliability of the Chinese version of the CES-D was 0.90 (Zhang et al., 2010).

During the experiment, the participants first filled in the general information questionnaire and the scale, and brachial blood pressure was taken approximately 30 min later using an electronic sphygmomanometer (U10K, Omron, Osaka, Japan). The participants were seated and relaxed during the measurement, and the center of the cuff was kept at the same height as the heart.

### Kinematic measurements

A force plate (P-6000, BTS bioengineering, Milan, Italy), 60*40 cm, with a sampling frequency of 1,000 Hz was used. It is composed of a pedal, sensor, and base. It provides three-dimensional dynamic data of the X-, Y-, and Z-axes and can display the COP data in real time. Data pre-processing and analysis software were written using MATLAB R2021a (Mathworks, Natick, MA, USA). The raw COP signals were filtered digitally with a 10 Hz low-pass filter (Butterworth). The sway area (mm^2^), total sway length (mm), mean velocity (mm/s), and amplitude (mm) of the COP in the anterior–posterior (AP) and medial–lateral (ML) directions were measured. Code or formula for calculation of COP (Table 1), for detailed COP parameters explanations, please see Quijoux et al. (2021).

**Table 1:** Description, code, and formula for calculation of COP.

Variable	Description	Code / formula
Sway area (95% confidence ellipse area)	The area of the ellipse which contains the true mean of (X_n_, Y_n_) 1 ≤ n ≤ N with a probability of 95%	2π * $\frac{N-1}{N-2}$ * F_0.95, 2, N -2_ * $\sqrt{RMS\;{\mathrm{ML}}^{2}*RMS\;{AP}^{2}-{COV}^{2}}$
Total sway length	Length of COP trajectory on the base of support	$\underset{n}{\sum }\sqrt{{\left(X_{n+1}-X_{n}\right)}^{2}+{\left(Y_{n+1}-Y_{n}\right)}^{2}\;}$
Mean velocity – AP	Determine how fast were the COP displacements	$\frac{\sum_{n=1}^{N-1}|X_{n+1}-X_{n}|}{T}$
Mean velocity – ML		$\frac{\sum_{n=1}^{N-1}|Y_{n+1}-Y_{n}|}{T}$
Amplitude – AP	Distance between the maximum and minimum COP displacement for AP and ML direction	max (COP_AP) − min (COP_AP)
Amplitude – ML		max (COP_ML) − min (COP_ML)

AP, anterior–posterior; ML, medial–lateral; X_n_, centered AP coordinates; Y_n_, centered ML coordinates; N, number of points of the signal; COV, covariance AP.

### fNIRS measurements and data processing

A multichannel near-infrared brain functional imaging device (NirSmart, Danyang Huichuang Medical Equipment Co., Ltd., Danyang, Jiangsu, China) was used to record brain activity. fNIRS is an optical method that uses a near-infrared spectral light source probe to noninvasively measure the concentrations of oxyhemoglobin (HbO_2_) and deoxyhemoglobin in the cerebral cortex. The system is composed of a near-infrared light source (LED) and an avalanche photodiode as detectors. The light source probe adopts wavelengths of 730 and 850 nm, and the sampling rate is 11 Hz. During the experiment, the participants wore a near-infrared headgear. The experiment used 20 light source probes and 20 detection probes to form 55 effective channels. The average distance between the transmitter and the detector was within 2.7–3.3 cm.

**Figure 1:**
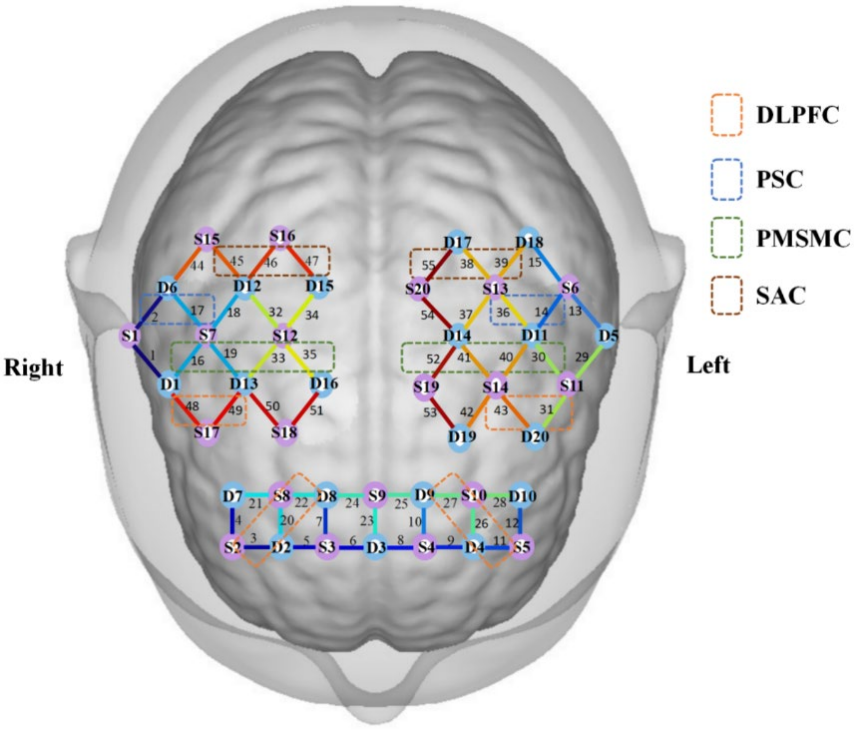
Brain regions and channels distribution map. S, sources; D, detectors.

Based on previous studies (Bolton, 2015; Fan et al., 2023; Herold et al., 2017a), the present study mainly observed the left and right primary somatosensory cortex (PSC), including channels 2/17/14/36; left and right premotor and supplementary motor cortex (PMSMC), including channels 16/19/30/33/35/40/41/52; left and right dorsolateral prefrontal cortex (DLPFC), including channels 3/11/20/22/26/27/31/43/48/49; and left and right somatosensory association cortex (SAC), including channels 38/39/45/46/47/55. The channel coordinates were taken from the reference standard international 10/20 electrode placement system. The obtained coordinates were converted into Montreal Neurological Institute (MNI) coordinates and further projected onto the MNI standard brain template using the spatial registration method of nirspace (Danyang Huichuang Medical Equipment Co., Ltd., Danyang, Jiangsu, China), as shown in Figure 1.

The NirSpark software (NirSpark, Danyang Huichuang Medical Equipment Co., Ltd., Danyang, Jiangsu, China) was used to process fNIRS data. First, the original light intensity data were converted into optical density data. Second, the spline interpolation method was used to remove the motion artifacts, which were mainly caused by the relative sliding between the scalp and the probe. Then, the original data were bandpass filtered in the range of 0.01–0.2 hz to remove physiological noise (such as breathing, cardiac activity, and low-frequency signal drift). Then, the denoised optical density data were converted into hemoglobin concentration data. Finally, the modified Beer–Lambert law was used to calculate the relative hemoglobin concentration changes of oxygen and hemoglobin (Herold et al., 2018). The average concentration of HbO_2_ within 5 s before the balance test began was calculated for the baseline correction on the signal to obtain the concentration change values of HbO_2_ (△HbO_2_) (Xu et al., 2024). The concentration of HbO_2_ in each task block was superimposed and averaged (Fan et al., 2023).

### Procedures

Standing balance control was performed in the Biomechanics Laboratory, School of Rehabilitation Medicine, Southern Medical University, Guangzhou, China. The balance function assessment of this study mainly included three balance tasks with different difficulties: task 1, standing with eyes open on a stable surface; task 2, standing with eyes closed on a stable surface, which can shield the participant’s vision; task 3, standing with eyes open on the surface of the foam pad, which can interfere with the proprioception of the participant. The specification of the foam pad (Airex Balance Pad Elite, Aili, Switzerland) was 50*41*6 cm. Standing on the foam pad can effectively reduce the input of body proprioception of participants (Hirase et al., 2015). Based on the pre-experiment of this study, when standing on the foam pad with eyes closed, the participants’ bodies swayed because of the interference of visual and proprioceptive inputs, showing a high risk of falls. To ensure the safety of the participants, this study does not involve the task of standing on the foam surface with eyes closed.

**Figure 2:**
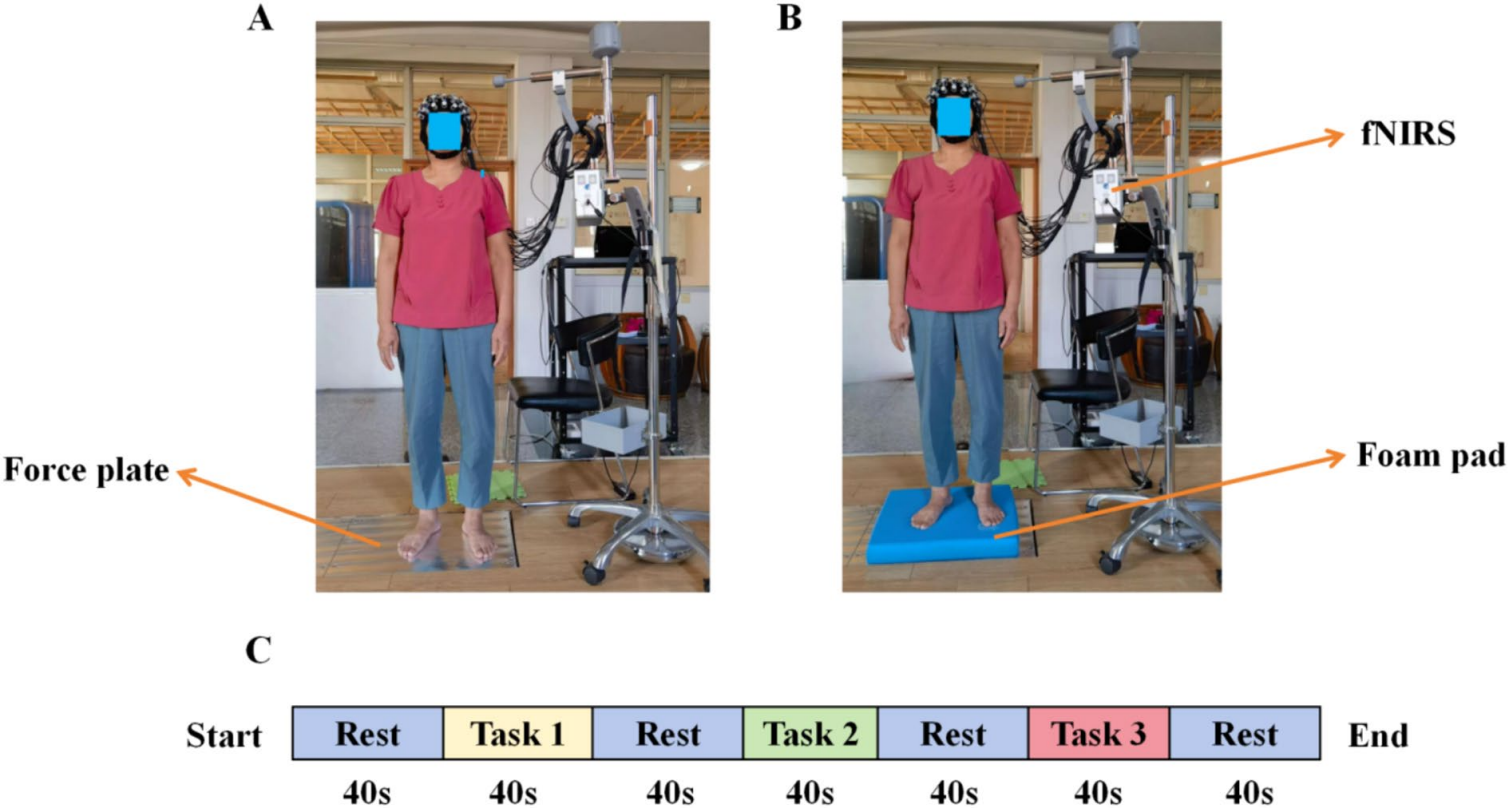
Experimental environment and block design. **(A)** task 1 and task 2, **(B)** task 3, and **(C)** study block design.

Participants maintained a natural standing posture, kept as still as possible, put their hands on both sides of the body, did not speak, and looked directly at mark point 1 m ahead (Xu et al., 2024). For foot placement requirements, when standing barefoot, the width of the feet can be freely selected, i.e., a comfortable position (Quijoux et al., 2020), as shown in Figures 2A,B.

Based on previous studies (Herold et al., 2017b; Lehmann et al., 2022; Lin et al., 2017), each type of standing task was tested three times, 40 s each time, the rest time is 40 s, and the results were taken as the average, as shown in Figure 2C.

### Statistical analysis

The Shapiro–Wilk and Levene tests were used to test the normality and variance homogeneity of the data to observe whether the hypothesis of parametric analysis was met. Data were presented as *n* (%) and mean (standard deviation) for categorical and normally distributed variables, respectively. When normality was reached, two independent sample *t*-test was used to test the presence of a significant difference in general information such as age, height, weight, body mass index (BMI), years of education, blood pressure and scales score, chi-squared test was used to test the presence of a significant difference in sex, covariance analysis was used to test the presence of a significant difference in cerebral cortex activation, COP sway area, total sway length, mean velocity, and amplitude between the two groups. Otherwise, a nonparametric test was considered. The False Discovery Rate method was used for multiple-comparison correction. According to the characteristics of the participants, the BMI and number of comorbidities were taken as covariates. All analyses were performed using IBM SPSS Statistics for Windows version 23 (IBM Corp., Armonk, NY, USA). *p* < 0.05 was considered statistically significant.

## Results

### Participant characteristics

The statistical results showed no significant difference between the two groups in sex, age, body height, body weight, years of education, fall history in the recent 1 year, CES-D, BBS, and ABC (*p* > 0.05). Significant differences in BMI, systolic blood pressure, diastolic blood pressure, number of comorbidities, and MMSE scores between the two groups (*p* < 0.05). In the HT group, 26 patients had grade 1 HT and 1 had grade 2 HT. No participants had grade 3 HT (Table 2).

**Table 2:** Characteristics of the participants (mean ± SD).

Variable	NT group (*n* = 30)	HT group (*n* = 27)	*p*-value
Sex (female/male)	15/15	15/12	0.792
Age (years)	69.30 ± 3.16	70.30 ± 4.07	0.304
Body height (cm)	162.42 ± 8.54	160.44 ± 6.00	0.323
Body weight (kg)	59.42 ± 10.34	61.13 ± 5.98	0.441
BMI	22.43 ± 2.81	23.73 ± 1.94	0.045
Years of education (years)	9.90 ± 2.94	9.56 ± 2.21	0.622
Fall history in recent year (times)	1.80 ± 0.41	1.89 ± 0.32	0.367
Number of comorbidities	0.70 ± 0.79	1.78 ± 1.12	<0.001
CES-D (maximum = 60)	4.90 ± 4.32	6.07 ± 3.32	0.259
Berg (maximum = 56)	55.17 ± 1.12	54.63 ± 1.55	0.136
ABC (maximum = 100)	92.35 ± 4.78	90.90 ± 3.80	0.213
Systolic blood pressure (mmHg)	124.63 ± 11.76	142.22 ± 17.80	<0.001
Diastolic blood pressure (mmHg)	76.40 ± 8.19	83.07 ± 10.01	0.008
Blood pressure classification (number of people)			
Grade 1	N/A	26	N/A
Grade 2	N/A	1	N/A
Grade 3	N/A	0	N/A
Course of hypertension (years)	N/A	10.70 ± 7.98	N/A

N/A, not applicable; Comorbidities, mainly hyperlipidemia and low back pain.

### Comparison of COP parameters between the groups

**Figure 3:**
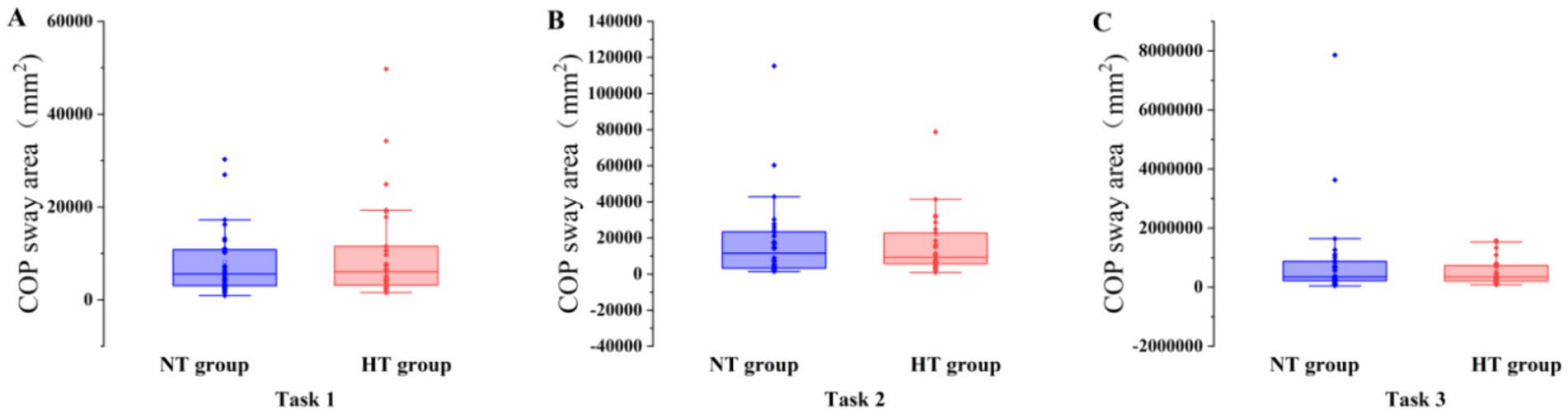
COP sway area in each task. The COP sway area of the two groups in **(A)** task 1, **(B)** task 2, and **(C)** task 3. Error bars indicate the minimum and maximum range.

**Figure 4:**
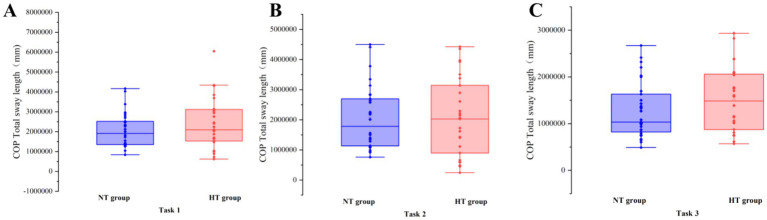
COP total sway length in each task. The COP total sway length of the two groups in **(A)** task 1, **(B)** task 2, and **(C)** task 3. Error bars indicate the minimum and maximum range.

**Figure 5:**
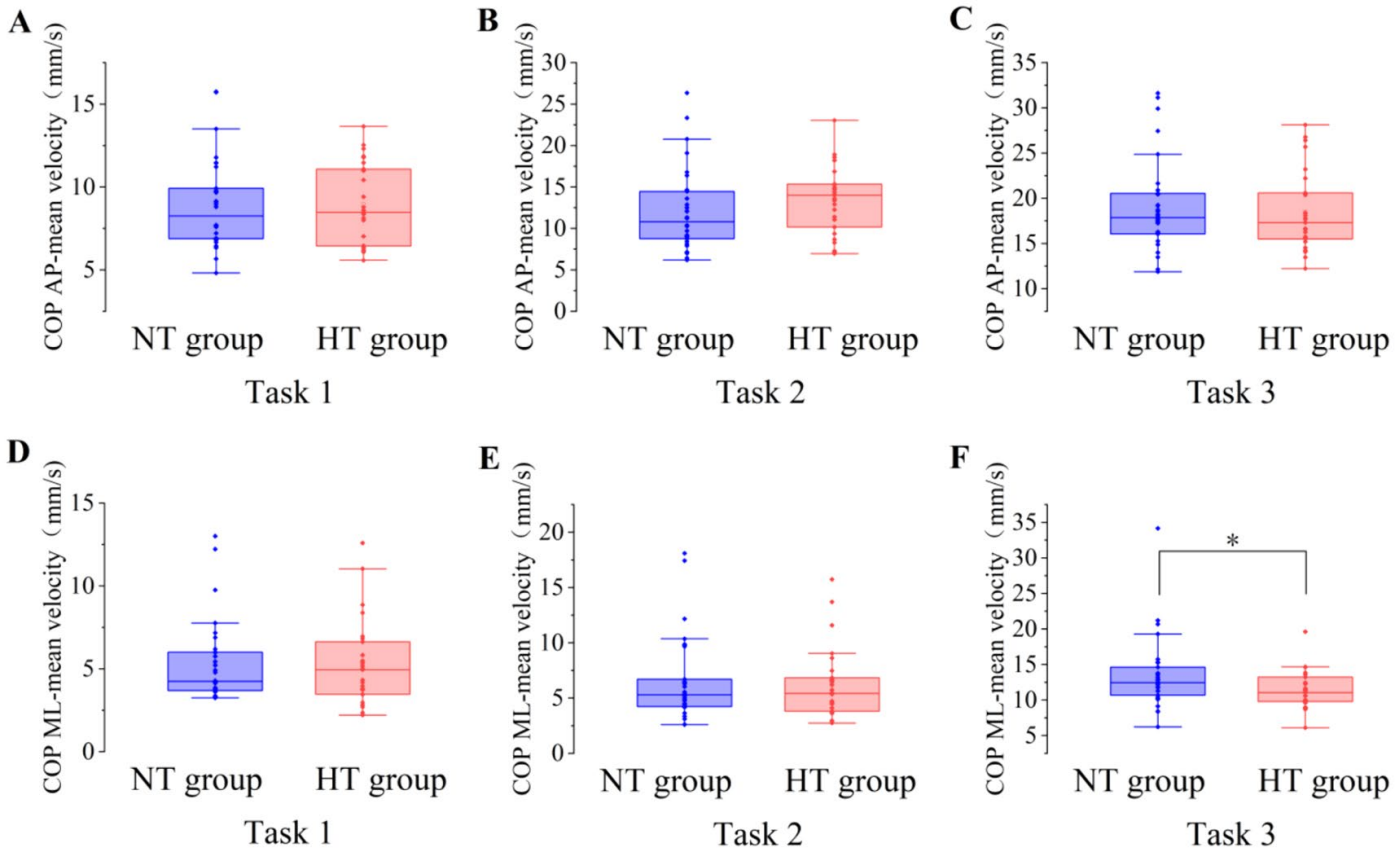
The mean velocity of the COP between the two groups in the AP direction in **(A)** task 1, **(B)** task 2, and **(C)** task 3, and the ML direction in **(D)** task 1, **(E)** task 2, and **(F)** task 3. AP, anterior–posterior; ML, medial–lateral. ^*^p < 0.05. Error bars indicate the minimum and maximum range.

**Figure 6:**
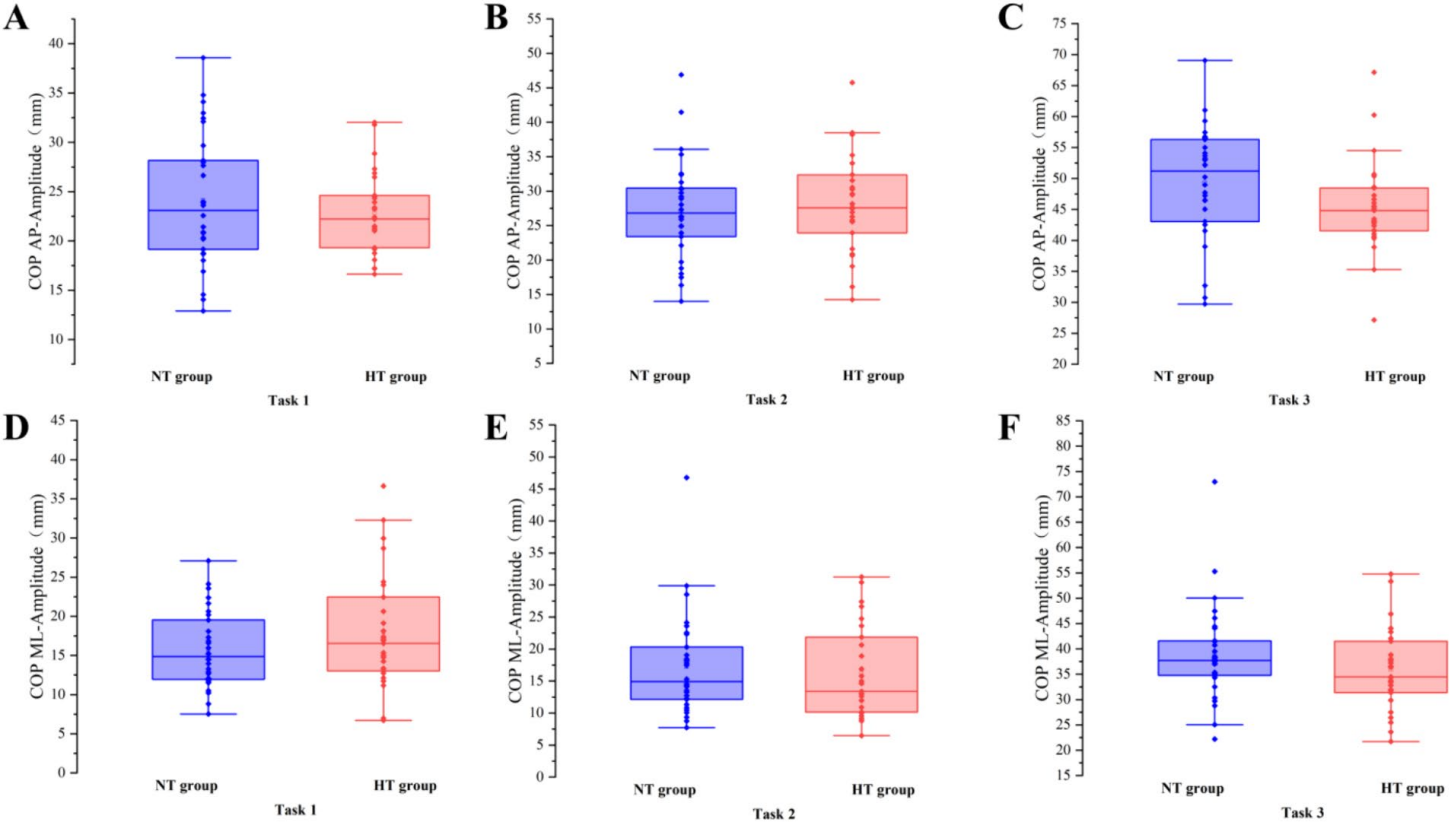
The amplitude of the COP between the two groups in the AP direction in **(A)** task 1, **(B)** task 2, and **(C)** task 3, and the ML direction in **(D)** task 1, **(E)** task 2, and **(F)** task 3. AP, anterior–posterior; ML, medial–lateral. Error bars indicate the minimum and maximum range.

Covariance analysis showed that at task 3, the mean velocity of the COP in the ML direction of the NT group was significantly higher than that of the HT group (*F* = 5.955, *p* = 0.018). No significant difference in the sway area, total sway length, mean velocity and amplitude of the remaining COP was found between the two groups (*p* > 0.05) (Figures 3–6).

### Intergroup cerebral cortex activation

**Figure 7:**
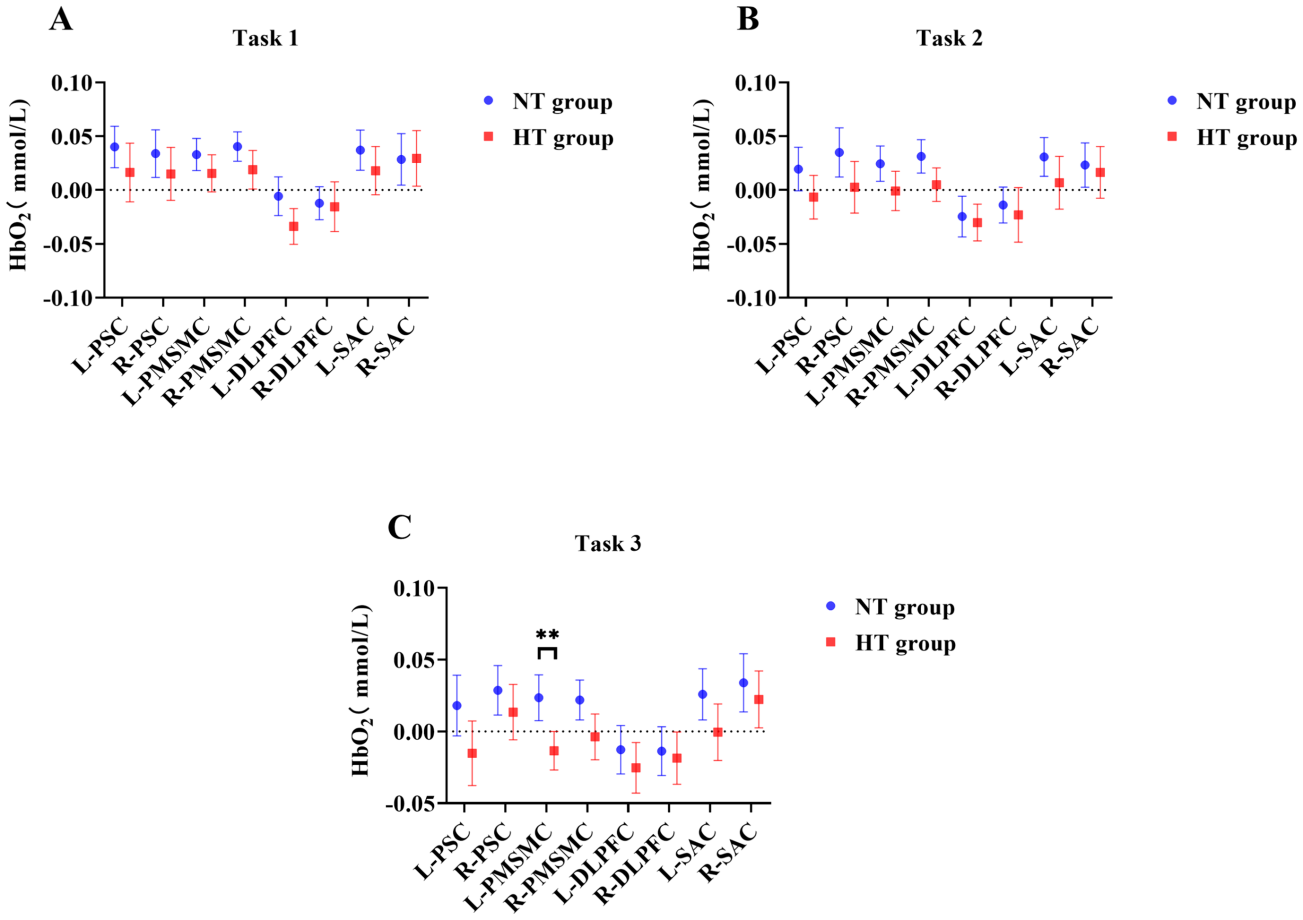
Comparison of cortical HbO_2_ activation in **(A)** task 1, **(B)** task 2, and **(C)** task 3 between the two groups. L-DLPFC, left dorsolateral prefrontal cortex; L-PMSMC left premotor and supplementary motor cortex; L-PSC, left primary somatosensory cortex; L-SAC, left somatosensory association cortex; R-DLPFC, right dorsolateral prefrontal cortex; R-PMSMC, right premotor and supplementary motor cortex; R-PSC, right primary somatosensory cortex; R-SAC, right somatosensory association cortex. ^**^p < 0.01. Error bars indicate 95% confidence interval.

**Figure 8:**
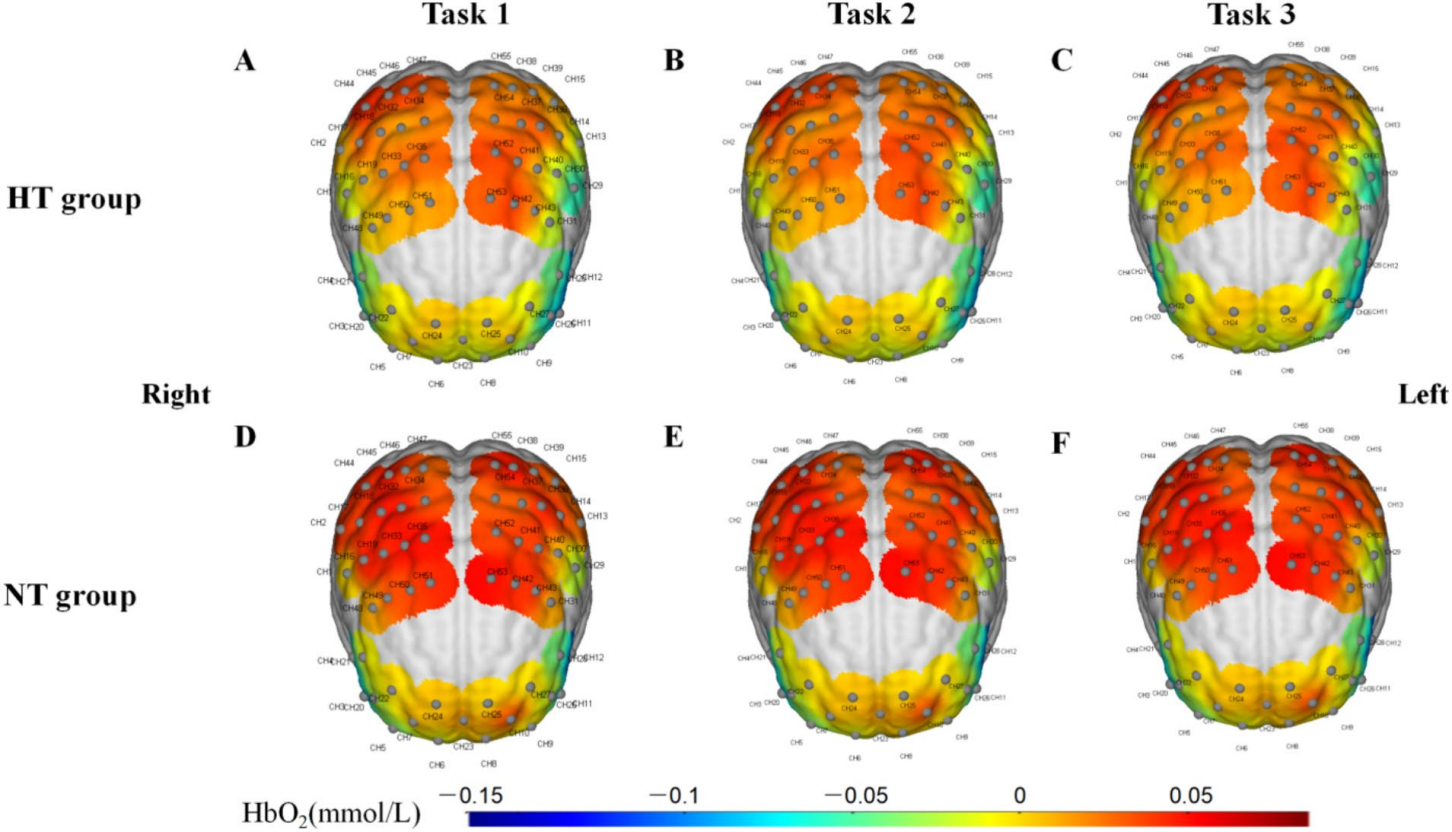
3D brain map of the activation of the cerebral cortex in **(A)** task 1, **(B)** task 2, and **(C)** task 3 in the HT group and **(D)** task 1, **(E)** task 2, and **(F)** task 3 in the NT group.

Covariance analysis showed that in task 3, the activation of the left premotor cortex and supplementary motor cortex (L-PMSMC) in the HT group was significantly lower than that in the NT group (*F* = 14.381, *p* < 0.001). No significant difference in cortical activation was found between the two groups under the remaining tasks (*p* > 0.05) (Figure 7). The 3D brain map of the cortical oxygenated hemoglobin activation under various task conditions is shown in Figure 8.

## Discussion

The current investigation compared the balance function and cerebral cortex oxygenation of older adults with HT and NT under different standing tasks. The findings partially support the low activation of the cerebral cortex in older adults with HT during challenging balance tasks.

This study found no difference in the sway area, total sway length, and amplitude of COP between the NT and HT groups when performing various standing balance tasks. When performing task 3, the mean velocity of the COP in the ML direction in the NT group was significantly higher than that in the HT group. Although this is inconsistent with the hypothesis of this study, this finding agrees with the results of a recent study, which shows that the data of the ML direction of the COP is more heterogeneous than that of the AP direction (Quijoux et al., 2020), suggesting that this result should be considered with caution. In this study, the results of the COP sway area, total sway length, mean velocity, and amplitude are basically consistent with those of Abate et al. (2009), which shows that in healthy older adults aged >65 years, the standing balance performance measured by the COP May not be related to HT. Thus, older adults with HT often complain about the symptoms of postural instability; however, they do not perform worse in the static posturography test. Meanwhile, no significant difference in the BBS and ABC scale scores was found between the two groups. This result further proves the lack of difference in balance function and activity balance confidence between older adults with HT and NT. The possible reason for these findings was that the older adults with HT in this study did not have complications and their blood pressure was well controlled (Abate et al., 2009). Further studies are needed to confirm our results.

This study indicated that the activation levels of the L-PMSMC in the HT group was significantly lower than that in the NT group when performing task 3, suggesting that when the proprioception of the HT group was disturbed, the HbO_2_ concentration was lower during this period. This result is in line with the findings of a previous study (Fitzgibbon-Collins et al., 2021), which revealed that a decline in cerebral oxygenation during standing in older adults was associated with postural instability, suggesting the impaired ability of older adults with HT to control their posture. Endothelial dysfunction, vasoconstriction, atherosclerosis of large vessels, and reduction in the number of small vessels in individuals with HT are the potential mechanisms (Muller et al., 2012; Triantafyllou et al., 2020). In addition, cerebral vascular abnormalities (e.g., white matter hyperintensity) are potential explanatory factors for the association between HT and physical function (Chen et al., 2022; Rosano et al., 2011). Work by Triantafyllou and colleagues observed minor increases in cerebral blood flow during challenging balance tasks for HT and not NT participants (Triantafyllou et al., 2020).

In our results, the HT group seems to show reduced oxyhemoglobin. Hypertension can lead to impaired neurovascular coupling, which means reduced blood flow during neuronal activity, resulting in an imbalance between the supply and demand of oxygen and metabolites in brain tissue (Iadecola and Gottesman, 2019; Phillips et al., 2016). Therefore, neurovascular coupling, as a mechanism in the brain that regulates cerebral blood flow, May explain our results, and future research needs to further confirm it.

In this study, only when proprioception was disturbed (i.e., task 3) that significant differences in cortical activation and COP were found between the two groups. This result is consistent with the finding of a previous study, which showed that if the inputs of the somatosensory systems are reduced through experiments and the main sensory inputs come from the vestibular system, the older adults have difficulty maintaining balance (Ruffieux et al., 2015). Premotor and supplementary motor cortices are related to motor preparation and monitoring (Puzzo et al., 2010). The results of this study also further showed that the L-PMSMC, as the related cortex of somatic movement (part of the sensorimotor cortex), was sensitive to standing balance tasks. Even if the COP sway area, total sway length, mean velocity and amplitude were not significantly different, changes in the cerebral cortex of older adults with HT who maintained challenging standing balance tasks were different, showing the characteristics of low activation. The possible reason was that the central changes occurred before COP parameters worsened in the impairment of standing balance function, which needs further research.

Some limitations of this study must be considered. First, the older adults with HT had grade 1 HT, and future studies must include older adults with higher-grade HT. Besides, given the limited sample size, this study did not analyze the effect of different antihypertensive drugs on the standing balance function of the HT group. Subgroup analysis of different antihypertensive drugs of older adults with HT can be carried out in the future to better control possible confounding factors. Lastly, the data of deoxyhemoglobin and total hemoglobin were not used for analysis in this study. It is useful to include the data of deoxyhemoglobin and total hemoglobin in the analysis in future research, as they help interpret the observed changes in HbO_2._

## Conclusion

The standing balance function of older adults with HT does not appear to be worse based on COP parameters than in those with NT. This study showed that the changes in the central cortex related to standing balance appear to be more indicative of balance control deficits in older adults with HT than changes in peripheral COP parameters, suggesting the importance of early measurement of cortical activation in older adults with HT at risk of falls. This study May provide guidance for the early detection of older adults with HT at risk of falls and the development of clinical rehabilitation strategies. Future studies on the early detection, early prevention, and early intervention of falls in older adults with HT are necessary.

## Data Availability

The raw data supporting the conclusions of this article will be made available by the authors, without undue reservation.
